# Genetic variants in red blood cell adhesion-related genes influence the severity of sickle cell anemia in a malaria-endemic region: Short title: Genetic variants in red blood cell adhesion-related genes in sickle cell anemia

**DOI:** 10.1007/s11033-026-12029-w

**Published:** 2026-05-28

**Authors:** Irina Matos, Brígida Santos, Elisângela Gonçalves, Pedro Lopes, Miguel Brito, Ana Paula Arez, Paula Faustino

**Affiliations:** 1 Instituto Nacional de Saúde Doutor Ricardo Jorge, I.P. (INSA), Avenida Padre Cruz, Lisbon, 1649-016 Portugal; 2 Centro de Investigação em Saúde de Angola (CISA), Caxito, Angola; 3 Hospital Pediátrico David Bernardino (HPDB), Luanda, Angola; 4 Global Health and Tropical Medicine (GHTM), LA-REAL, Instituto de Higiene e Medicina Tropical (IHMT), UNL, Lisbon, Portugal; 5 ESTeSL- Escola Superior de Tecnologia da Saúde, H&TRC- Health & Technology Research Center, Instituto Politécnico de Lisboa, Lisbon, Portugal; 6 Research Centre for Anthropology and Health (CIAS), Department of Life Sciences, University of Coimbra, Coimbra, Portugal; 7 Laboratório Associado TERRA, Instituto de Saúde Ambiental (ISAMB), Faculdade de Medicina da Universidade de Lisboa, Lisbon, Portugal

**Keywords:** Sickle cell anemia, Malaria, Angola, Genetic modifiers, *ICAM-1*, *CD36*

## Abstract

**Background:**

Sickle cell anemia (SCA) is a genetic disease marked by abnormal hemoglobin S and sickle-shaped red blood cells. It is highly prevalent in sub-Saharan Africa, especially in Angola, where SCA and malaria are major causes of childhood mortality. This study aimed to explore whether genetic variants in genes associated with red blood cell adhesion to the vascular endothelium influence the manifestations of SCA in Angolan pediatric patients in the context of malaria.

**Methods and results:**

The study enrolled 65 pediatric SCA patients living in Luanda or Caxito. Their clinical, hematological, and biochemical profiles were monitored through longitudinal pediatric follow-up appointments. Fifteen polymorphic sites were genotyped in *CD36* and *ICAM-1* genes using PCR, Sanger sequencing, and fragment analysis by capillary electrophoresis. Malaria infection was evaluated by detecting *Plasmodium* species DNA through PCR analysis of blood spot samples. The *CD36* variant rs3211891_C is revealed for the first time as a potential modulator of anemia severity in SCA. Additionally, the *CD36* variant rs3211938_G, along with the *ICAM-1* variants rs5491_T and rs5496_A, significantly impacted the severity of the hematological phenotype in SCA. Furthermore, SCA patients carrying the *ICAM-1* rs5494_T variant showed a 5.63-fold increased risk of having malaria infection compared to those with the wild-type genotype.

**Conclusions:**

This study enhances our understanding of genetic modifiers of red blood cell adhesion to the vascular endothelium and their influence on the severity of pediatric SCA in the context of frequent concomitant malaria infection in Angola.

## Introduction

Sickle Cell Anemia (SCA) is an autosomal recessive genetic disease caused by the mutation c.20 A > T in the *HBB* gene, which leads to the synthesis of the abnormal hemoglobin S (HbS). When deoxygenated, HbS polymerizes inside red blood cells (RBCs), inducing cellular deformation into a sickle shape. The sickled RBCs are stickier and less deformable than normal, contributing to vascular occlusion, chronic hemolysis, and severe anemia [1, 2]. SCA is one of the most common hereditary diseases in the world. The incidence is estimated to be between 300,000 and 400,000 neonates globally each year, the majority in sub-Saharan Africa [3]. In this region, the disease is associated with an extremely high mortality rate, particularly in children under 5 years of age [3].

The coexistence of SCA and malaria in Africa suggests strong evolutionary selection pressures, maintaining the HbS allele at high frequencies. Unlike sickle cell carriers (HbAS) who are protected against severe malaria, homozygous individuals (HbSS) infected with malaria tend to develop very severe anemia and are highly susceptible to its lethal effects [4, 5]. Both malaria and SCA are associated with high destruction of RBCs and widespread systemic inflammation [6] and even low-level infections can precipitate severe anemic crises that would likely prove fatal without rapid intervention [7]. Recently, it was shown that the protective effect of HbS against severe malaria varies greatly according to the parasite genotype, which suggests that malaria parasites have evolved to evade HbS protection [8].

When *Plasmodium falciparum* infects the human host, it undergoes a complex life cycle, infecting liver cells before invading RBCs, where it multiplies asexually. The pathology of severe malaria is largely driven by the parasite ability to modify infected RBCs (iRBCs), leading to their adhesion to the vascular endothelium. A key factor in this process is the *P. falciparum* Erythrocyte Membrane Protein 1 (PfEMP1), a highly variable surface protein encoded by the *var* gene family. PfEMP1 facilitates cytoadherence by binding to host endothelial receptors, allowing iRBCs to sequester in capillary beds, and avoiding splenic clearance. This sequestration contributes to microvascular obstruction, endothelial activation, and inflammation, leading to complications such as cerebral malaria, placental malaria, and severe anemia [9, 10]. Two of the most well-characterized host receptors for PfEMP1 are the Cluster of Differentiation 36 (CD36) and the Intercellular Adhesion Molecule 1 (ICAM-1) [11], both of which also play crucial roles in SCA pathophysiology.

CD36 is a class B scavenger receptor found on monocytes, macrophages, platelets, immature red blood cells (reticulocytes), and endothelial cells. It plays a role in fatty acid processing, inflammatory pathways, and immune system functions [12]. In malaria, CD36 serves as a major adhesion receptor for iRBCs expressing PfEMP1, facilitating their accumulation in post-capillary venules and contributing to the development of severe malaria symptoms [13]. In SCA, CD36 is involved in both sickled RBCs and reticulocytes adhesion to the vascular endothelium [14, 15]. Certain *CD36* gene variants have been associated with reduced protein expression, potentially altering RBCs adhesion and influencing SCA and malaria severity [reviewed in 16]. Individuals with *CD36*-null mutations, which are more common in African populations, exhibit reduced PfEMP1-mediated adhesion, potentially altering the severity of malaria and SCA-related vascular complications [16, 17].

ICAM-1 is a membrane-bound glycoprotein found on endothelial cells, white blood cells (WBC), and other immune cells. It is crucial for mediating inflammation and guiding the movement of leukocytes to sites of immune activity [18]. Like CD36, ICAM-1 is a major receptor for PfEMP1, facilitating the binding of iRBCs to the microvascular endothelium. Increased ICAM-1 expression is linked to cerebral malaria, where iRBCs accumulate in brain capillaries, causing neurological complications [19]. In SCA, ICAM-1 is involved in the adhesion of WBC to activated endothelium, notably contributing to inflammation. Moreover, studies have shown that sickled RBCs stimulate ICAM-1 expression in cultured human endothelial cells. This upregulation leads to higher levels of ICAM-1 on the cell surface, along with increased release of its soluble form - both of which are associated with a greater risk of vaso-occlusive crises [20]. Additionally, in SCA, the adhesion of sickled RBCs is closely linked to severe hemolysis and the presence of right-to-left shunts within the heart or lungs [21]. Also, sickled RBCs interact with activated neutrophils and platelets, forming extracellular aggregates that can adhere to the vessel walls, resulting in the obstruction of the blood microcirculation. Therefore, the elevated expression of ICAM-1 on endothelial cells, its increased binding affinity for sickled RBCs, and its key involvement in oxidative stress and inflammation, position it as a major contributor to the severity of SCA [20–22].

Given that genetic variations in genes encoding adhesion molecules can significantly influence the clinical outcomes of SCA and, in malaria-endemic regions, affect susceptibility to malaria, investigating common polymorphisms in at-risk populations is crucial for developing targeted interventions - especially in countries like Angola, where both conditions present serious public health concerns [23, 24]. This study, therefore, aimed to investigate the impact of genetic polymorphisms in *CD36* and *ICAM-1* on SCA in pediatric patients from Angola. Understanding these associations may offer valuable insights into disease management and support the development of targeted therapeutic strategies and public health initiatives to reduce the burden of SCA in a malaria endemic region.

## Materials and methods

This study is part of a larger longitudinal investigation conducted within an Angolan pediatric cohort with SCA, involving Hospital Pediátrico David Bernardino in Luanda, Hospital Geral do Bengo, and the Centro de Investigação em Saúde de Angola (CISA) in Caxito. The main objective of the primordial study was to clinically characterize children with SCA and assess their response to hydroxyurea (HU) treatment [23]. For the specific investigation presented here, which focuses on the impact of genetic modifiers of blood cell adhesion on SCA severity in the context of malaria, 65 children were enrolled during the pre-HU phase, given that HU treatment is known to alter clinical manifestations of the disease. Inclusion criteria comprised Angolan ancestry, age between 3 and 12 years, and a confirmed diagnosis of SCA. Exclusion criteria were recent blood transfusion, previous HU treatment, active tuberculosis, HIV infection, neoplasia, and bone marrow dysfunction. Informed consent was obtained from the children legal guardians. The study received ethical approval from the Angolan Ministry of Health as well as from all participating institutions.

### Patients’ clinical evaluation

A schedule of regular medical appointments was established for the 65 children with SCA. At the initial visit, the pediatrician performed a comprehensive physical examination and conducted a structured anamnesis, gathering demographic data and current symptoms. Retrospective clinical information was obtained from hospital records, including details of the initial SCA presentation and occurrences of splenomegaly, hepatomegaly, and jaundice. After this first consultation, patients were followed up every three months for a period of six to nine months.

At each consultation, approximately 4 mL of peripheral blood was collected from each patient using EDTA and dry serum tubes. These samples were utilized for hematological and biochemical analyses to establish the baseline clinical and laboratory profile, reflecting the steady-state condition of SCA patients not yet treated with HU. Additionally, drops of peripheral blood were applied onto Guthrie cards (WHAT10531018 Card Protein Saver, 903^®^ Whatman™) for subsequent extraction of human and malaria parasite DNA.

### Hematological and biochemical characterization

Peripheral blood samples were analyzed in a XT-2000i Hematology Analyzer (Sysmex, Japan) to measure standard hematological parameters, including hemoglobin (Hb) concentration, RBC count, mean corpuscular volume (MCV), mean corpuscular hemoglobin (MCH), red cell distribution width (RDW), reticulocyte count, WBCs, neutrophils, and platelets. Biochemical markers of hemolysis, such as lactate dehydrogenase (LDH) and total bilirubin, were assessed using Cobas C11 (Roche, Switzerland). For each patient, hematological and biochemical values were calculated as the average of at least three measurements taken during steady-state periods, defined as being afebrile, without blood transfusion, or vaso-occlusive episodes in the preceding three months.

### Human and parasite DNA extraction and malaria infection molecular diagnosis

The patients’ blood spots collected on Guthrie cards were used to extract human and parasite DNA using the saponin/Chelex-100 method as described [25]. DNAs were stored at -80 °C until analyzed. To detect and identify *Plasmodium* spp., a PCR amplification of the small subunit ribosomal RNA (ssrRNA) genes was performed following the protocol established by Snounou et al. [26]. Specific identification of *Plasmodium ovale* was carried out using a nested PCR method according to Fuehrer et al. [27].

### *CD36* and *ICAM-1* genotyping

Human genomic DNA concentrations were measured using the NanoDrop One Spectrophotometer (ThermoFisher Scientific Inc., Waltham, MA, USA). Fifteen polymorphic sites were genotyped in *CD36* and *ICAM-1* genes. In *CD36* gene, six single nucleotide variants (SNVs) located at gene promoter (rs1984112 and rs1413661), intron 4 (rs3211891 and rs3211892), exon 5/intron 5 (rs3211893), and exon 10 (rs3211938) were characterized by Sanger sequencing after amplification of three DNA fragments of 314, 707 and 390 bp, respectively. PCR conditions and primers sequences were already described [28, 29]. Automated Sanger sequencing reactions were performed using the BigDye Terminator v 1.1. Cycle Kit (Applied Biosystems) in a Biometra Thermocycler. Fluorescent signals and strand sizes were differentiated through capillary electrophoresis in the automated sequencer 3500 Genetic Analyzer (Applied Biosystem), and results were analyzed using FinchTV v1.4.0 software (Geospiza, Inc.). Moreover, a short tandem repeat (STR) located at intron 3 of *CD36* gene, Ins(TG)n, rs3138813, was characterized by PCR using a FAM fluorescent-tagged forward primer [30], followed by PCR product separation and sizing in the 3500 Genetic Analyzer (Applied Biosystem) with the Gene Mapper Software 6 along with the appropriate molecular weight marker, GeneScan TM 500 LIZ^®^ Size Standard. To confirm these results, five DNAs identified as homozygous for (TG)_11_, (TG)_12,_ (TG)_13,_ (TG)_16,_ and (TG)_17_ were Sanger sequenced and then used as quality control samples.

In *ICAM-1* gene, eight SNVs were analyzed, located at exon 2 (rs5491), intron 2 (rs5030352), exon 4 (rs1799969 and rs5494), exon 5 (rs1801714), intron 5 (rs5496), and exon 6 (rs5497 and 5498). These polymorphic regions were amplified in two PCR fragments of 416 pb (containing exon 2 and intron 2), and 1053 bp (from exon 4 to exon 6). Primers’ sequences and PCR conditions were already reported [31, 32]. PCR fragments were sequenced by Sanger approach, as described above.

### In silico analysis

Population allele and genotype frequencies were queried in Ensembl website (www.ensembl.org), Ensembl release 113 [33]. The varSeak bioinformatic tool (https://varseak.bio/) was used to study variants affecting intronic sequences for potential disruption of splicing donor/acceptor sites, which could influence mRNA sequence and stability, as well as protein expression.

### Statistical analysis

The statistical analysis was done using IBM SPSS Statistics software (version 27). For descriptive statistics, continuous variables were shown as means with standard deviations, medians, and their minimum and maximum values. To check if the data followed a normal distribution, the Shapiro-Wilk test was used. Since none of the quantitative variables were normally distributed, non-parametric tests like Mann-Whitney-Wilcoxon and Kruskal-Wallis were used for comparisons. For categorical variables, contingency tables were created, and the Chi-square or Fisher’s exact test was applied. A *p*-value less than 0.05 was considered statistically significant. Association between genetic variants and clinical, blood, and biochemical parameters were assessed through dominant and codominant genetic models.

## Results

### Population description – SCA clinical, hematological and biochemical phenotypes

This study was performed on 65 Angolan pediatric SCA patients who had never taken HU, aged between 3 and 12 years, 32 males and 33 females. A baseline hematological and biochemical profile was established for each SCA patient based on the average of several laboratorial determinations in steady state conditions. Furthermore, patients’ SCA complications and SCA first manifestation information were collected from their healthcare records. The description of these data is presented in Table 1.

**Table 1:** Demographic data, laboratory parameters, and clinical manifestations of the SCA patients studied

Patients’ characteristics	No.	(%)	Mean	SD	Median	Min	Max
**Gender**							
Male	32	49.2					
Female	33	50.8					
**Age** (years old)			6.9	2.8	7.0	3	12
**Hematological Parameters**							
Total Hemoglobin (g/dL)	65		7.3	0.9	7.2	5.5	9.9
Red Blood Cells (x10^12^/L)	65		2.9	0.5	2.8	2.1	4.8
Mean Corpuscular Volume (fL)	65		77.6	7.2	78.3	60.3	97.3
Mean Corpuscular Hemoglobin (pg)	65		25.6	2.6	25.8	18.8	30.6
Reticulocytes (%)	65		10.0	3.8	9.6	3.1	25.6
Platelets (x10^9^/L)	65		444.1	152.6	451.0	171.2	770.7
Leucocytes (x10^9^/L)	65		13.9	4.3	13.2	7.2	29.7
**Biochemical Parameters**							
Lactate Dehydrogenase (U/L)	57		428.1	123.4	423.8	246.3	682.9
Total Bilirubin (mg/dL)	63		1.3	0.9	1.1	0.2	5.1
**Clinical manifestations**							
Hepatomegaly	27	41.5					
Splenomegaly	9	13.8					
Jaundice	32	49.2					
**SCA first manifestation**							
Dactylitis	43	66.2					
Vaso-occlusive pain	18	27.7					
Severe anemia	4	6.2					

No. = number of patients; SD = standard deviation

On average, children showed their first signs of the disease at 16 months of age. Dactylitis was the most common initial symptom, occurring in 66.2% of cases, followed by vaso-occlusive pain crises (27.7%) and severe anemia (6.2%). Other typical clinical features observed included jaundice (32 patients, 49.2%), hepatomegaly (27 patients, 41.5%), and splenomegaly (9 patients, 13.8%). As anticipated, these children exhibited chronic hemolytic anemia, characterized by reduced RBC counts (2.9 ± 0.5 × 10¹²/L) and Hb levels (7.3 ± 0.9 g/dL), along with elevated serum markers of hemolysis such as LDH (428.1 ± 123.4 U/L) and total bilirubin (1.3 ± 0.9 mg/dL). Moreover, they also have high reticulocyte count (10.0 ± 3.8%), which is an indirect biomarker of hemolysis, reflecting increased erythropoietic activity in response to anemia and chronic hemolysis. Furthermore, their mean platelet count was 444.1 ± 152.6 × 10⁹/L, which is consistent with reactive thrombocytosis, often observed in SCA due to bone marrow compensation for anemia.

### Genetic findings in *CD36* and *ICAM-1* genes

Fifteen genetic variants, 14 SNVs and one STR were characterized in *CD36* and *ICAM-1* genes. In the *CD36* gene, the genotyped regions included the gene promoter, exons 5 and 10, and introns 3, 4 and 5. For the *ICAM-1* gene, the genotyped regions comprised exons 2, 4, 5 and 6, and introns 2 and 5. The allele and genotype frequencies of the SNVs are presented in Table 2. Results were compared with those reported by the 1000 Genomes Project Consortium, phase 3, Ensembl released 113 [33].

**Table 2:** Single nucleotide variants studied in *CD36* and *ICAM-1* genes and the corresponding genotype and allele frequencies

Gene	Genetic variant	Type of variant	Genotype	No.	Genotype frequency (%)	Allele frequency (%)
*CD36*	c.-184 + 11,225 A > G (rs1984112)	intronic	AA	35	53.8	A − 74.6 G − 25.4
			AG	27	41.5	
			GG	3	4.6	
	c.-184 + 11308G > C (rs1413661)	intronic	GG	3	4.6	G − 30.8 C − 69.2
			GC	34	52.3	
			CC	28	43.0	
	c.282-31T > C (rs3211891)	intronic	TT	49	75.0	T − 85.4 C − 14.6
			TC	13	20.0	
			CC	3	5.0	
	c.282–10 A > G (rs3211892)	intronic	AA	8	12.3	A − 26.9 G − 73.1
			AG	19	29.2	
			GG	38	58.5	
	c.429 + 2T > C (rs3211893)	intronic	TT	60	92.3	T − 94.6 C − 5.4
			TC	3	4.6	
			CC	2	3.0	
	c.975T > G (rs3211938)	nonsense	TT	48	74.0	T − 86.9 G − 13.1
		p.Tyr325*	TG	17	26.0	
			GG	0	0	
*ICAM-1*	c.167 A > T (rs5491)	missense	AA	33	50.8	A − 70.8 T − 29.2
		p.Lys56Met	AT	26	40.0	
			TT	6	9.2	
	c.331 + 39 C > G (rs5030352)	intronic	CC	0	0	C − 18.7 G − 81.3
			CG	24	37.0	
			GG	41	63.0	
	c.721G > A (rs1799969)	missense	GG	65	100	G − 100 A − 0
		p.Gly241Arg	GA	0	0	
			AA	0	0	
	c.846 C > T (rs5494)	synonym	CC	51	78.5	C − 88.5 T − 11.5
		p.Asp282=	CT	13	20.0	
			TT	1	1.5	
	c.1055 C > T (rs1801714)	missense	CC	63	96.9	C − 98.5 T − 1.5
		p.Pro352Leu	CT	2	3.1	
			TT	0	0	
	c.1181-12 G > A (rs5496)	intronic	GG	56	86.2	G − 92.3 A − 7.7
			GA	8	12.3	
			AA	1	1.5	
	c.1190G > A (rs5497)	missense	GG	52	80.0	G − 90.0 A − 10.0
		p.Arg397Gln	GA	13	20.0	
			AA	0	0	
	c.1405 A > G (rs5498)	missense	AA	55	84.7	A − 91.5 G − 8.5
		p.Lys469Glu	AG	9	13.8	
			GG	1	1.5	

No. = number of patients

**Fig. 1:**
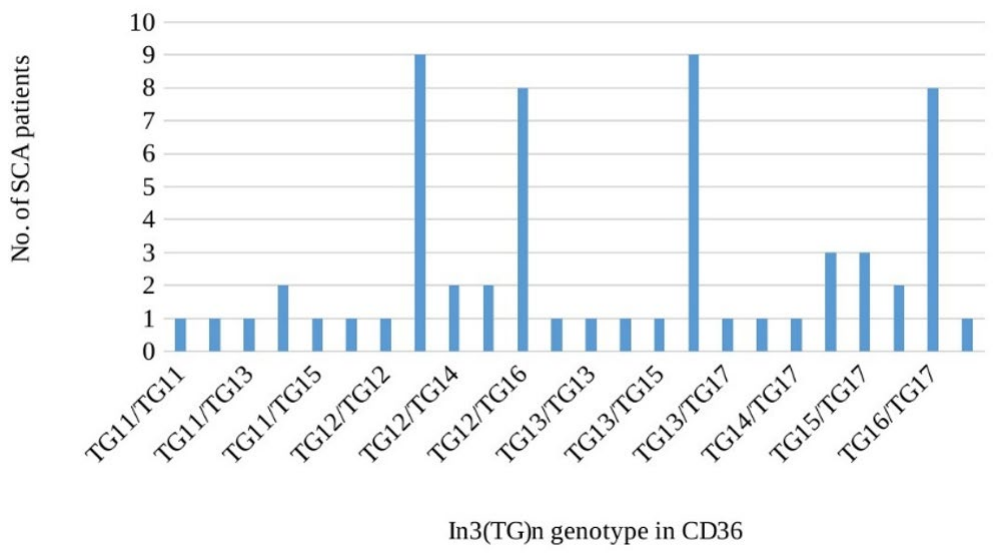
Genotypes of the short tandem repeat In3(TG)n in *CD36* gene detected in the SCA studied patients. (TG)n represents the number of repetitions of the dinucleotide TG

Regarding the STR in *CD36*, In3(TG)n, rs3138813, amplification failed in three DNA samples, so valid results were only obtained for 62 patients. Seven different alleles were found, varying from (TG)_11_ to (TG)_17_, and 24 different genotypes (Fig. 1). The most frequent allele found was the (TG)_16_, (33 alleles, 26,6%), followed by the (TG)_12_, (25 alleles, 20,2%) and the (TG)_13_, (24 alleles, 19,4%). Concerning genotypes, the most frequent were the (TG)_12_/(TG)_13_ and the (TG)_13_/(TG)_16_, both found in nine patients, and the (TG)_12_/(TG)_16_ and the (TG)_16_/(TG)_17_, both found in eight patients. Only 1.6% of our patients have the considered wild type genotype (TG)_12_/(TG)_12_ [30]. The STR allele and genotype frequencies found in Angolan SCA patients studied here are significantly different (*p* < 0.001) from what is reported in Genome Aggregation Database (gnomAD) v4.1, Ensembl released 113 [33] for the African/African American population, where the (TG)_12_ has an allele frequency of 82.0% and (TG)_13_ of 18.0%.

### Association of genetic variants with hematological, biochemical, and clinical phenotypes

Association studies were conducted between the patients’ genotypes and the average hematological and biochemical parameters. Both dominant and codominant genetic models were used, with statistically significant findings presented in Table 3.

**Table 3:** *CD36* and *ICAM-1* genotype associations with hematological and biochemical parameters in the studied SCA patients

Genetic variant	Genotype	No.	Hb (g/dL)	*p*	RBC (x10^12^/L)	*p*	LDH (U/L)	*p*	Retic. (%)	*p*	Plat. (x10^9^/L)	*p*
**rs3211891** **in** ***CD36***	TT	49	7.43	**0.013**	2.93	0.074	431.93	0.457	9.65	0.403	457.03	0.328
	TC	13	7.20		2.90		435.92		10.57		391.15	
	CC	3	5.98		2.32		344.02		12.37		461.30	
**rs3211938** **in** ***CD36***	TT	48	7.26	0.737	2.82	0.293	430.83	0.739	10.37	0.154	468.94	**0.030**
	TG + GG	17	7.47		3.09		419.34		8.81		373.78	
**rs5491** **in** ***ICAM-1***	AA	33	7.54	0.098	3.05	**0.044**	389.64	**0.020**	9.13	0.109	463.39	0.104
	AT + TT	32	7.08		2.83		465.05		10.82		424.11	
**rs5496** **in** ***ICAM-1***	GG	56	7.21	**0.009**	2.84	**0.036**	425.73	0.694	10.31	**0.044**	442.67	0.954
	GA + AA	9	7.99		3.20		440.13		7.79		452.63	

No. = number of patients; the *p*-values highlighted in bold are statistically significant

Association studies on *CD36* variants have identified a significant relationship between the rs3211891T > C polymorphism and Hb levels. Individuals with the wild-type TT genotype had an average Hb level of 7.43 g/dL, while those homozygous for the C allele (CC) exhibited a significantly lower Hb level of 5.98 g/dL (*p* = 0.013). This suggests that the rs3211891_C allele is associated with worsened anemia. In the same way, RBCs count was lower in CC homozygotes (2.32 × 10¹²/L) compared to TT (2.93 × 10¹²/L), although not statistically significant (*p* = 0.074). Another variant in *CD 36* gene, the rs3211938T > G, seems to have a potential role in negatively modulating thrombocytosis in SCA patients, as the group of patients who have this variant (TG and GG genotypes) showed lower platelet counts (*p* = 0.030) contrasting with the wild-type group.

Regarding the association studies performed with variants in *ICAM-1* gene, we have observed that the rs5491A > T variant has a role in worsening the hemolytic anemia, as the group of patients who have this variant (genotype AT and TT) shows higher LDH levels (465,05 U/L) and lower RBC counts (2.83 × 10¹²/L) than the wild-type group (*p* = 0.020 and *p* = 0.044, respectively). On the contrary, the rs5496G > A variant in *ICAM-1* seems to have an important role on improving anemia, as evidenced by a higher Hb concentration of 7.99 g/dL and increased RBC count of 3.20 × 10¹²/L observed in SCA patients who have the A variant (GA and AA genotypes) compared to the wild-type GG group (*p* = 0.009 and *p* = 0.036, respectively). In addition, this variant was also associated with lower reticulocyte count (7.79%) compared with wild type (10.31%), (*p* = 0.044), likely a result of reduced demand for stimulation of erythropoiesis.

### Characterization of malaria infection cases and association with *CD36* and *ICAM-1* genotypes

Molecular diagnosis of *Plasmodium* species was performed in only 54 out of the 65 SCA patients due to missing appointments, withdrawals and resorting to other health units during emergency situations. *Plasmodium* infection was identified in seven of the 54 SCA patients (13%), having been the *P. falciparum* identified in six cases and *P. malariae* in one case. We examined whether the genotypes of SCA patients in *CD36* and *ICAM-1* were associated with the detected malaria-positive cases. The rs5494_T variant in the *ICAM-1* gene was found at a high frequency in the group of SCA patients with malaria infection. Specifically, 30.8% of SCA patients carrying the T allele (CT or TT genotypes) revealed positive for malaria infection, compared to only 7.3% of patients with the wild-type CC genotype. This indicates that individuals with the T allele have a significantly 5.63 times higher risk, of malaria infection compared to those with the CC genotype (*p* = 0.028; OR = 5.63, 95% CI: 1.07–29.73; Table 4).

**Table 4:** *ICAM-1* genotype association with malaria infection in SCA patients

Genetic variant	Genotype	Malaria infected SCA patients No. (%)	Non-infected SCA patients No. (%)	*p*	Odds Ratio	95% CI
rs5494 in *ICAM-1*	CC	3 (7.3)	38 (92.7)	0.028	5.63	1.07–29.73
	CT + TT	4 (30.8)	9 (69.2)			

CI = Confidence Interval

## Discussion

In Africa, both SCA and malaria are prevalent diseases, and their interaction over the ages highlights the intricate connection between human genetics, infections, and evolutionary processes. Human genetic diversity has played a key role in shaping the patterns of susceptibility to malaria and the severity of SCA. Attending to the hypotheses that sickled RBCs, iRBCs, and other blood cells adhesion to the vascular endothelium mediated by CD36 and ICAM-1 have an important role in the variability of both SCA and malaria, we characterized several SNV and one STR within *CD36* and *ICAM-1* genes and searched for association with anemia severity, level of hemolysis, and malaria infection in pediatric Angolan SCA patients. All children were homozygous for the *HBB*:c.20 A > T mutation and exhibited chronic hemolytic anemia, which, in several cases, adversely affected organs such as the spleen and liver, resulting in splenomegaly and hepatomegaly, respectively. Additionally, we observed that the elevated rate of intravascular hemolysis in these patients led to increased serum bilirubin levels, which subsequently accumulated in tissues and caused jaundice. Furthermore, a compensatory response from the bone marrow was evident, characterized by enhanced erythropoiesis and an increased release of reticulocytes into the bloodstream. Moreover, this population exhibited elevated platelet, and leukocyte counts which may reflect underlying inflammatory processes associated both with hemolytic anemia and vaso-occlusive events, commonly observed in SCA, as well as with immune response to infectious diseases, highly prevalent in tropical regions [34, 35].

*CD36* is a highly polymorphic gene, with various nucleotide variants occurring both within and outside the coding regions. Some of them can reduce CD36 expression level, alter the extracellular ligand-binding domain, or even cause protein deficiency, which may affect CD36 function and have been associated with some diseases [16]. In our study, among the positive associations identified between genotypes and phenotypes, we observed that the SNV rs3211891 (c.282-31T > C), located in intron 4 of the *CD36* gene, was associated with lower Hb level and reduced RBC count, which suggests that this variant may contribute to increase anemia severity in patients with SCA. In fact, this variant is located only four nucleotides upstream of the branch point site of the fourth intron, which is essential for the binding of the spliceosome machinery and, thus, important for signaling the splicing acceptor site of the intron. It could be hypothesized that the variant is disturbing the splicing mechanism and, consequently, this intron may be retained in the mRNA and become coding, altering the CD36 protein. A review of the literature revealed limited research on the consequences of the rs3211891 variant, and to our knowledge, it has not previously been reported in association with disease modulation. Thus, our findings may represent the first evidence suggesting a potential role for this variant in modulating anemia severity in SCA.

Still in the *CD36* gene, the G allele of the SNV rs3211938 (c.975T > G), was found with a frequency of 13.1%. This nonsense mutation is located in exon 10 of the *CD36* gene and gives rise to a premature stop codon, p.Tyr325Ter. It is known that mRNAs containing premature stop codon trigger the nonsense-mediated decay (NMD) mechanism to prevent the synthesis of truncated proteins [36]. Numerous nonsense mutations have been identified in the CD36 gene, and notably, these mutations are especially prevalent in certain African populations from regions where malaria is endemic [37]. Due to nonsense mutations, certain types of cells will not express CD36 on their surface, which may suggest a protective effect against severe manifestations of infectious disease but the absence of CD36 and its consequences on malaria severity remain controversial and have yet to be fully elucidated [17, 37, 38]. Furthermore, some authors have suggested that the lack of CD36 expression on reticulocytes, mature RBCs, and sickled RBCs does not alter the clinical course of patients with SCA [39]. Nevertheless, in our study, SCA patients who have this nonsense variant tend to have less anemia and hemolysis and a significant lower level of platelets count (*p* = 0.030), when compared with the wild-type genotype (TT), which denotes some benefits at hematological level. The low expression of CD36 in patients with SCA may indeed be related to a smaller increase in platelet levels as CD36 plays an important role in platelet activation and the inflammatory response. Conversely, the low expression of CD36 may also affect other cellular functions. In the literature, this variant in the *CD36* gene has been linked to several biological functions, including fatty acid metabolism and lipid homeostasis regulation, even in patients with SCA [40, 41]. However, its overall impact on the health of SCA patients requires further investigation.

Our results concerning the characterization of the (TG)_n_ repeat in the *CD36* gene (rs3138813) revealed a wide diversity of genotypes, with the canonical (TG)_12_ repeat occurring at low allele and genotype frequencies. According to the literature, both the (TG)_12_ and (TG)_11_ repeats produce the wild-type *CD36* transcript that confer protection against cerebral malaria in a Thai population [30]. In contrast, other repeat lengths result in an abnormal transcript due to the skipping of the fourth and fifth exons, producing a truncated CD36 isoform that likely affects the binding affinity of iRBC [30]. However, these findings have not been consistently validated across different populations, and as well, no association was observed between this polymorphism and malaria episodes in our SCA cohort.

Considering the *ICAM-1* gene, the missense c.167 A > T variant, rs5491, p.Lys56Met, was originally described in Kilifi, Kenya, as predisposing to cerebral malaria [42]. However, this association was not validated in other African regions and there is conflicting evidence indicating that the mutation may either increase resistance to malaria or contribute to a greater risk of severe disease, depending on the context of the mutation and other genetic or environmental factors [43, 44]. The interaction between PfEMP-1 and ICAM-1 involves the region containing the Kilifi mutation, and the differences in susceptibility to cerebral malaria are likely to be due to variations in the binding strength and affinity of different PfEMP-1 variants expressed by various parasite strains, as well as how these variants interact with ICAM-1 polymorphisms [43]. Furthermore, another modulatory effect may arise from the binding of the blood protein fibrinogen to ICAM-1, an interaction that is completely interrupted by the presence of the ICAM-1 Kilifi variant [45]. Consequently, it has been proposed that fibrinogen may enhance malaria susceptibility in individuals who are homozygous for the ICAM-1 Kilifi mutation [45]. On the other hand, and concerning SCA, a study has shown that ICAM-1 mediates the adhesion of sickled RBCs in a patient-specific manner, with stronger adhesion correlating with severe hemolysis and the presence of right-to-left cardiac or pulmonary shunts [21]. In agreement, our results concerning the ICAM-1 Kilifi variant revealed that patients with the AT or the TT genotype experienced a significant worsening of their hemolytic anemia, but no association with malaria cases was found.

The *ICAM-1* rs5496 (c.1181-12G > A) intronic variant, located in the splice polypyrimidine tract of intron 5, is likely to affect *ICAM*-1 splicing and expression. We have found that in SCA patients, the presence of the variant (GA + AA genotypes) was associated with a more favorable hematological profile, including higher Hb levels (*p* < 0.001), increased RBC count (*p* = 0.036), and reduced reticulocyte percentage (*p* = 0.044), suggesting reduced hemolysis and lower erythropoietic stress (Table 3). Therefore, this SNV has proven to be a beneficial genetic factor for several hematological and biochemical manifestations of the SCA. In conclusion, the two *ICAM-1* variants discussed above (rs5491 and rs5496) appear to have distinct functional consequences on the severity of hemolytic anemia in SCA. Their effects may be independent, as they are unlikely to be in strong linkage disequilibrium, given that they are located approximately 9.9 kb apart from each other in the GRCh38 human reference genome. Thus, independent associations for these variants are biologically plausible; however, confirmation will require further studies in larger cohorts, as well as functional and haplotype analyses.

Our results concerning another *ICAM-1* variant, rs5494, c.846 C > T, a synonym variant located at exon 4, revealed a significant association with the detected positive cases of malaria (Table 4). Effectively, our results revealed that the group of SCA patients presenting the variant (genotype CT or TT) is 5.63 times more likely to have malaria infection than the group of SCA patients with the wild type genotype (CC). Since it is a synonymous variant, p.Asp282=, not altering the structure of the protein, it is possible that its effect is made at the regulatory level of *ICAM-1* gene expression. However, the underlying pathophysiological mechanism remains unknown. A study published in the literature reports an association between this variant, along with three additional SNVs in the *ICAM-1* gene, and malaria endemicity in Africa [46]. Our findings are consistent with these observations and support the need for further research into the potential role of this variant in malaria susceptibility.

An important strength of our study is that none of the enrolled SCA patients had previously been treated with HU, making this a HU-naïve SCA cohort. This fact is not always taken into account in this type of genotype-phenotype association studies. In fact, HU exerts a range of effects, including anti-inflammatory actions, increased fetal Hb production, reduced expression of endothelial adhesion molecules, nitric oxide release, and decreased levels of reticulocytes, leukocytes, and platelets [47, 48]. Therefore, we are confident that the genotype-phenotype significant associations identified in this study are not biased by HU treatment. However, we consider the limited sample size to be a key limitation of this study, and our findings should therefore be interpreted as preliminary evidence of a potential role of adhesion molecule gene polymorphisms in modulating SCA severity and malaria susceptibility. We emphasize the exploratory nature of this work and the need for validation in larger, independent cohorts.

On the other hand, one of the study’s objectives was to evaluate malaria episodes in Angolan children with SCA. However, several challenges hindered this investigation, including missed follow-up visits, participant withdrawal, and the use of alternative healthcare facilities during emergency situations [23]. These limitations underscore the need for a follow-up study with a larger sample size and more robust monitoring to confirm the findings reported here and to better elucidate the relationship between *CD36* and *ICAM-1* genotypes and malaria susceptibility in pediatric SCA patients in Angola.

## Conclusion

Although SCA and malaria are highly prevalent diseases in Africa, studies on the relationship between the two are scarce. This work explored the relationship between SCA and malaria in African children, focusing on genetic modifiers related to blood cell adhesion to vascular endothelium. The research identified key variants in the *CD36* and *ICAM-1* genes that influence anemia severity, hemolysis, and malaria infection in pediatric SCA patients in Angola. Specifically, the *CD36* variants rs3211891_C and rs3211938_G, as well as *ICAM-1* variants rs5491_T and rs5496_A, are significantly modulating the severity of the hematological phenotype of SCA. Moreover, the *ICAM-1* rs5494_T variant was found associated with malaria infection in these SCA children. Our results emphasize the need for further research in African populations to confirm these findings and explore their impact on other clinical manifestations of SCA and malaria infection. Understanding these genetic factors could lead to targeted therapies for managing SCA complications, particularly those related to sickled RBCs adhesion the endothelium, in the context of malaria endemic regions.

## Data Availability

Due to the sensitive and potentially identifiable nature of the clinical data (including medical history and records), and in accordance with the informed consent provided by participants, these data cannot be made publicly available. The molecular data supporting the conclusions of this study are fully presented within the main text.

## Abbreviations

CD36: Cluster of Differentiation 36
CISA: Centro de Investigação em Saúde de Angola
DNA: Deoxyribonucleic acid
EDTA: Ethylenediamine tetraacetic acid
Hb: Hemoglobin
*HBB*: Beta-globin protein coding gene
HbS: Hemoglobin variant S
HU: Hydroxyurea
ICAM-1: Intercellular Adhesion Molecule 1
iRBCs: infected Red Blood Cells
LDH: Lactate Dehydrogenase
MCH: Mean Corpuscular Hemoglobin
MCV: Mean Corpuscular Volume
mRNA: Messenger Ribonucleic Acid
PCR: Polymerase Chain Reaction
PfEMP1: *Plasmodium falciparum* Erythrocyte Membrane Protein 1
RBC: Red Blood Cell
RDW: Red Cell Distribution Width
SCA: Sickle Cell Anemia
STR: Short Tandem Repeat
SNVs: Single Nucleotide Variants
ssrRNA: small subunit ribosomal RNA
WBC: White Blood Cells
